# Empirical Evidence for Professional Practice and Public Policies: An Exploratory Study on Social Exclusion in Users of Primary Care Social Services in Spain

**DOI:** 10.3390/ijerph16234600

**Published:** 2019-11-20

**Authors:** Víctor M. Giménez-Bertomeu, Yolanda Domenech-López, Miguel A. Mateo-Pérez, Nicolás de-Alfonseti-Hartmann

**Affiliations:** Department of Social Work and Social Services, Faculty of Economics & Business, University of Alicante, 03690 Alicante, Spain; yolanda.domenech@ua.es (Y.D.-L.); ma.mateo@ua.es (M.A.M.-P.); nicolas.dealfonseti@ua.es (N.d.-A.-H.)

**Keywords:** social exclusion, intensity, scope, typologies, professional practice, policy making, quality of life

## Abstract

This study examines the social exclusion characteristics of a sample of users of primary care social services in two local entities in Spain. The objective of this study was to identify the intensity and scope of social exclusion in an exploratory way and to look at the typology of existing exclusionary situations to inform policy making and professional practice. Data from 1009 users were collected by primary care social services professionals, completing the Social Exclusion Scale of the University of Alicante (SES-UA). The dimensions with the greatest levels of social exclusion in the study population were those related to work/employment, income and education and training. The dimensions with an intermediate level of exclusion were those related to housing and social isolation. Social acceptance, family and social conflict and health were the dimensions with the lowest levels of exclusion. The analysis also showed the existence of five significantly different groups, that showed five different life trajectories along the continuum between social exclusion and social inclusion. The results show the importance and utility of developing professional and policy intervention protocols based on research evidence, with the objective of improving the quality of life of the users.

## 1. Introduction

### 1.1. Social Exclusion

Social exclusion is “a broad term that refers to the inability of certain groups or individuals to participate fully in society” [1] (p. 1). It has been defined as a ‘‘dynamic multidimensional processes driven by unequal power relationships interacting across four main dimensions—economic, political, social and cultural—and at different levels including the individual, household, group, community, country and global levels”. Social exclusion exists across a continuum of inclusion/exclusion characterized by unequal access to resources, capabilities and rights, which leads to health inequalities” [2] (p. 2). 

In this way, social exclusion is a “a gradual process of progressive exclusion from a situation of social integration made up of various stages of intensity that go from precariousness or mild vulnerability to grave situations of social exclusion” [3] (p. 184). This refers to a situation of “being excluded (leaving out) (process of self-exclusion/exclusion) that affects certain people and social groups” [4] (p. 18).

The phenomenon of social exclusion brings together three fundamental characteristics [3,5,6,7,8]:

- Structural origin: The causes are structural, derived from inequalities generated by the socioeconomic, political and cultural model.
- Multidimensional character: Social exclusion affects multiple dimensions or facets of people’s lives over time.
- The dynamic nature of the process: Social exclusion is not static, rather the intensity and scope vary with the life cycle of people over time.

**Figure 1:**
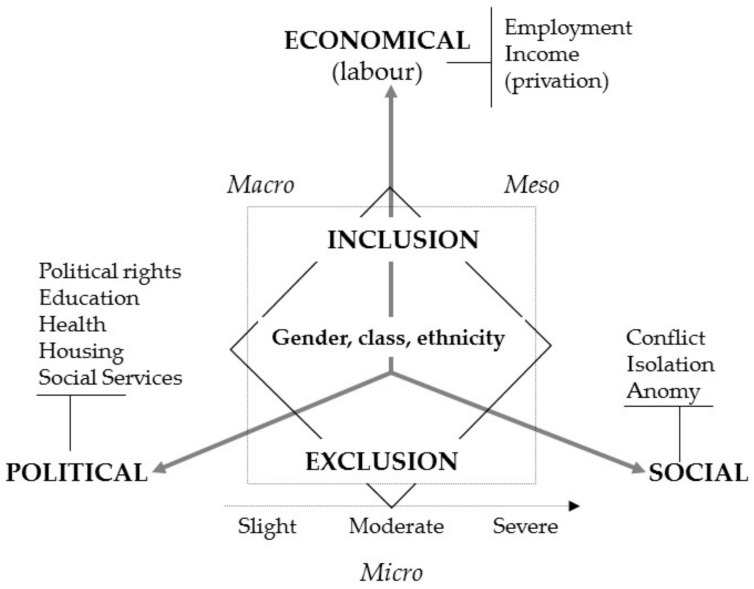
Axes and aspects of social exclusion. Source: [14] (p. 191).

Although the definition of social exclusion presents nearly as many levels as authors who have approached the topic, the majority of the work on social exclusion establish a series of “dimensions” or “areas of influence” by which the processes of social exclusion directly affect people (Figure 1). Therefore, social exclusion is not a “state” in which people/households or groups are immersed, rather it is a group of processes linked to the life cycle that are a result of broader structural factors. Some authors signal the importance of individual/personal factors [9] or factors related to the life cycle of life course in terms of understanding the consequences of social exclusion [10,11,12,13]. Without forgetting the importance of individual factors, it is important to point out the structural character of exclusion, generated by different types of unequal social relationships. The individual suffers from the consequences of these relationships at all levels.

Said another way, processes of social exclusion prevent satisfying human needs in certain sociocultural contexts and times. These processes can directly affect people in any stage of life, preventing the development of individual capacities [15,16,17]. They limit or make more difficult wellbeing and certainly affect the full development of quality of life, understood as one’s personal level of desired well-being. These processes are multidimensional, with etic (universal) and emic (culture-bound) properties and with objective and subjective components, influenced by personal and environmental factors [18,19].

In addition, social exclusion is variable in its scope (it can manifest in terms of the accumulation of problems in different areas of life) and in its intensity (problems in these areas can manifest with different levels of severity). The interaction between the two aspects of exclusion means that it can appear due to severe problems in a single area, or it can be the result of a multitude of less severe problems in various areas. In this way, from the point of view of time, it can be of short or long duration [11,12].

In Spain in recent years, different empirical research has been carried out that is quantitative in nature and has provided interesting aspects related to the measurement of the prevalence of social exclusion in our country [20]. In this way, a range of different approaches is available related to the measurement of social exclusion [21].

In general, empirical studies begin with a conceptual discussion and establish an operative definition of social exclusion that considers that in practice, social exclusion is a property of people and as such, can be seen objectively and measured through questioning and observation of the study cases/subjects. Later, different dimensions of exclusion are established (economic, political, and social dimensions), with indicators/variables constructed for each. The indicators can come from secondary sources (surveys carried out by official statistical organizations, which can be harmonized or not, such as the European Panel of Households, and the Quality of Life Survey, among others). They can also come from primary sources (that is, studies that design their own questionnaires and indicators on exclusion, given that the objectives or operational approach on which the study is based are different that those established by official statistical organizations).

### 1.2. Social Services and Social Exclusion

Many times, social intervention must present simple options to address complex problems, such as is the case of research on social exclusion [22].

It is precisely in the public social services system that simple and appropriate instruments are needed for the analysis of social exclusion processes. It is necessary that they provide for planning, implementation and evaluation of social insertion itineraries. This analytical approach is not incompatible with the relational dimension of social workers in their professional labor with users. 

In terms of social exclusion, the methodological challenge of primary care social services in the context of intervention lies precisely in analyzing the assessment carried out with users, structural processes and tailoring the social intervention to aspects that in many cases are the consequences of social exclusion and not is causes. 

In this sense, determining the factors of exclusion to which people are exposed and analyzing the prevalence of their consequences is an indispensable task that must accompany personal development and promotion of the active inclusion of people in social services. This also implies recognizing the role and utility of research as one of the sources of knowledge for social intervention, combined with other sources of knowledge (organizations, practitioners, users and policy community) to build knowledge useful for social care [23].

Furthermore, the increasing development of social services care devices also inevitably leads to the standardization of certain actions, especially when they affect a considerable number of people that must be addressed in an equal way [24]. In our opinion, assessment of social exclusion is one of these actions that is susceptible to being standardized in part through rigorous and systematic use of instruments for data collection. It should be mentioned that there is a necessary personalization of responses with the participation of the users in the assessment. 

The development of a tool for data collection and systematization of social exclusion situations is a key element to achieve the role assigned to social services of “social support for incorporation” with the accompaniment of personal development [24]. Public social services need to adapt assessment and intervention of exclusion profiles, because exclusion has a different impact throughout the life cycle on people and because the processes that it generates vary across time [25]. It is in this process of adaptation, improvement and adjustment that the different professionals of social intervention (especially social workers) can and should provide their experiences and knowledge and become actively involved in processes of research, systematization of information and analysis. 

This work has an exploratory and descriptive orientation. It aims to provide empirical evidence to municipal social services about the social exclusion situation of users, in terms of the scope (affected areas and dimensions that present deficits or difficulties) and the typology of situations of social exclusion. These are designed to be useful for professional practice and for policy makers, given that knowledge of the scope of social exclusion permits identification of the areas in which it is necessary to develop an intervention; knowledge of the intensity of exclusion allow for establishing priorities in terms of action that take into account the gravity of the affectation in each area; knowledge of the typologies of exclusion situations allows for designing interventions and planning different policies for each type of these typologies. Certainly, the aim is to improve the quality of life of the users that social services providers work with.

## 2. Materials and Methods 

### 2.1. Participants

Data were collected from 1009 users of primary care social services from the county councils of Osona and Valles Oriental (Barcelona, Spain).

Anonymous data on the cases were randomly collected by 126 social services workers from different basic areas of social services from both counties, of which 88% were women and 12% men, social workers (64%) and social educators (36%). 

The inclusion criteria of the cases were:

- People served by professionals (with direct interaction and not through a third party) in the first care services during the data collection period.
- People between 18 and 65 years old and emancipated minors (16 years or older).

To guarantee randomness and, at the same time, make data collection compatible with workloads, professionals selected cases following different procedures:

- If data from all cases could be collected in a single day, they followed the order of appointment.
- If the above could not be done, they collected select cases cited on different days and in different time slots.

Given the mode of selection of the cases and the exploratory purpose of the work, the sample is not generalizable to the set of users of the Social Services of the county councils. However, such a large sample allows us to describe the scope and intensity of the exclusion of this population.

### 2.2. Instrument

Professionals provided data on the selected cases by completing the Social Exclusion Scale of the University of Alicante (SES-UA), a measuring instrument developed by the authors to systematize data on the scope and intensity of the social exclusion of users of primary care social services. Using as a frame of reference the sources selected in the bibliographic review of recent publications on the concept and measurement of exclusion (2008–2015), the SES-UA was developed from (1) various operational proposals for measuring social exclusion/inclusion [5,6,26,27,28,29,30] and (2) secondary sources that had useful exclusion indicators to make their measurement operational (EU 2020 Strategy, National Statistics Institute, Survey of Living Conditions, Active Population Survey, Population and Housing Census), (3) sources used by the county councils for the collection of sociodemographic and administrative data and (4) other sources [31].

The objective of the SES-UA is to establish the position of a person along the inclusion/exclusion continuum, which provides information to the professional about whether the person is in a situation of inclusion, in a situation of risk of exclusion or vulnerability, or in a situation of social exclusion. The instrument can be used with people between 18 and 65 years old and with emancipated minors (16 years or older).

The SES-UA is structured into six areas (housing/accommodation, economy, labor, education/training, health and social relationships); eight dimensions (housing/accommodation, economic situation/income level, employment situation, education and training, health, isolation, family and social conflict and social acceptance); 23 sub-dimensions and 40 indicators.

The instrument classifies each situation analyzed into four categories, according to the intensity of the exclusion and the dimension assessed: no exclusion, slight exclusion, moderate exclusion or severe exclusion.

### 2.3. Procedure 

For the data collection, an online questionnaire was designed that collected the data of the SES-UA, administrative data (basic area of social services, file number and person in the family unit to which the instrument was applied, code and professional profile of the evaluator, date of data collection) and sociodemographic family data of the user (municipality, county and province of residence, date of birth, gender, nationality, legal status in the country, belonging to a ethnic minority, level of education, type of illness/disability, need for health care and follow-up, number of people in the family unit, and number of family unit members between 0 and 16 years old).

Data were collected between March and May 2016.

For their aggregate statistical processing, data were anonymized.

### 2.4. Ethical Issues

Before data collection, informed consent was obtained from professionals to participate in the study. They also received a training session on the manual and use of the instrument about on the instructions for data collection. 

With respect to cases, the professionals required prior consent from the users selected, and the public organizations participating provided their data anonymously in accordance with Organic Law 15/1999 on the protection of personal data, which provides that consent required for the cession of personal data to third parties is not required when the cession occurs between public administrations for the objective of later data use for reasons of historical, statistical or scientific nature (art.11.2.e).

### 2.5. Data Analysis

Data analysis included univariate, bivariate and multivariate analysis.

According to the social work literature on data analysis [32,33,34,35]: descriptive statistics were used to summarize characteristics of participants, and mean and standard deviation were used to describe users’ characteristics measured by continuous variables.

The data on the sociodemographic profiles of the sample were compared to the same data referring to the general population in the Municipal Register of Inhabitants (2015) and in the Population and Housing Census (2011) in order to identify the similarities and differences between the participants in comparison to the general population of the territorial area studied

The aforementioned analyzes were complemented with k means clustering to provide empirical evidence on the groups in which participants could be classified according to the extent and intensity of the exclusion. This type of analysis allows a population to be classified into a number of groups defined by the researcher. For this purpose, using the ordinal scores of each case in each of the eight dimensions of the SES-UA as a classification variable, a different number of groups was tested until the most satisfactory classification was obtained. This was at the discretion of the research team, who determined a classification into five groups. This number of groups has also been identified in previous work related to the typology of trajectories between social exclusion and social inclusion [36]. The cluster centers of each group and the sociodemographic characteristics of each cluster were analyzed. 

This analysis was intended to serve the social services professionals of the participating organizations in terms of decision-making by establishing links between the descriptive results of the instrument (scope and intensity of social exclusion) and subsequent professional intervention, and to provide evidence for the development of a future typology of insertion itineraries that could be developed by the professionals

It is important to note that this analysis was exploratory since it was carried out with data from participants and not from a statistically representative sample of the population served by county social services. In the future, this work could be complemented by confirmatory analysis that uses a representative sample of the population.

All statistical analyses were conducted using SPSS, Version 24.0 (Armonk, NY, USA).

## 3. Results

### 3.1. Sociodemographic Profile

Participants’ age ranged from 16 to 65 years (Mean = 41.72; SD = 10.82). Nearly two-thirds were women (65%) and a third were men (35%). The number of family members ranged between 1 and 9 members (Mean = 3.14; SD = 1.60). The majority of users made up part of family units with minors between 0–16 years of age (64%) (Mean = 1.94; SD = 098). Spanish was the primary nationality (61%) and not belonging to the Roma ethnicity (97%). Most had a regular legal status in the country (96%). Around 58% had not completed required education levels, compared to 41% who had. The majority did not present illness or disability (66%) nor limitations on ability (75%). About 61% did not need care and/or health follow-up, and 26% needed it in a punctual way. Finally, they resided in towns of around 6000 inhabitants or less (30%), about 22% lived in towns with 6001–15,000 inhabitants, 29% in towns with 15,001–30,000 inhabitants and 19% in towns with over 30,000 inhabitants. 

Comparison of the sociodemographic data from the sample compared with the general population showed that:

- Women made up 15% more of the sample than the general inhabitant population (50.1%).
- The study population was over-represented in terms of those aged 25 to 29 (with differences between 0.5% and 6%), while for the rest of the intervals (minors under age 25 and those over age 59) it was under-represented (with differences of 2.4% and 5.8%).
- The family units with five or more members were over-represented in the study population with respect to the Census (with differences between 0.1% and 8.4%), and especially units with five and six members. The family units with one to four people were under-represented (with differences between 0.6% and 10.9%).
- In the sample, there was 29% greater foreign population than the resident population.

### 3.2. Scope and Intensity of Social Exclusion

Social exclusion of the participants has the following characteristics (Table 1):

1. Dimensions with greatest levels of exclusion are:
  - D3—Work. Severe exclusion affects 67%, and moderate exclusion affects 10%. Both are present in 77% of the total.
  - D2—In terms of the economic situation/income level, severe exclusion affects 58% of people and moderate exclusion affects 24%. Together they make up 81% of the total.
  - D4—In terms of education and training, severe exclusion affects 44% and moderate exclusion affects 33%. Both are present in 77% of the total.
2. The dimensions in which the majority of people do not present social exclusion or present slight exclusion are, in the following order:
  - D1—Housing. About 56% of people do not present social exclusion in this dimension. Moderate exclusion affects 20% and severe exclusion affects 10%. Together they represent 30% of the total. Fourteen percent find themselves with slight exclusion. Those affected by some level of social exclusion make up 44% of the total.
  - D6—Isolation. Although 32% of people do not present exclusion in this dimension, one in four presents moderate exclusion (25%), and severe exclusion affects 13%. Together they make up 38% of the total. Slight exclusion is around 30%. Thus, people affected by some level of exclusion represent 68% of the total.
3. The dimensions with the least level of exclusion, or those in which the majority of the population does not present exclusion (with percentages over 65% of the total are (from lower to higher levels)):
  - D8—Social acceptance. Severe and moderate exclusion affected 28% of the population. There were no situations of slight exclusion.
  - D7—Family and social conflict: Severe and moderate exclusion affected 29% of the population, and slight exclusion affected 2%.
  - D5—Health. Severe exclusion and moderate exclusion affected 35% of the population. There were no observed situations of slight exclusion.

**Figure 2:**
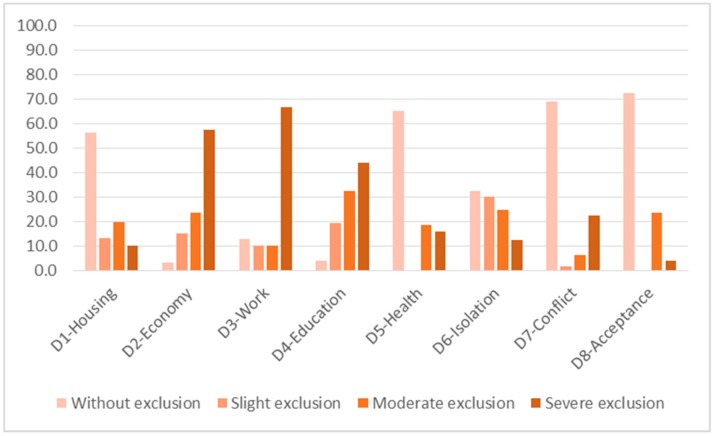
Intensity and scope of social exclusion (%).

Graphically represented, the level of social exclusion (intensity) in the study population for each of the dimensions (scope) is shown in Figure 2:

### 3.3. Classification of the Population: Trajectories

We carried out a cluster analysis with five groups according to the number of the five typologies identified by other authors in previous work [36]. We used non-hierarchical k means clustering using as classification variables the ordinal scores for each of the eight dimensions of the SES-UA, which classify the intensity of the exclusion along a scale from 1 (without exclusion) to 4 (severe exclusion). 

The iteration process used for the classification of the cases ended after ten iterations.

The results of the Analysis of Variance (ANOVA) showed the existence of five groups with means that were significantly different in all of the dimensions (Table 2).

In terms of the distribution of the study population in each cluster (Table 3), the greatest part was concentrated in cluster 3, followed by clusters 2 and 1. In clusters 4 and 5, the number of cases was similar.

The general characteristics of the clusters, accounting for the scope and intensity of the observed exclusion, are the following (Table 4):

- No cluster coincides in terms of the intensity and exclusion in a single dimension.
- Dimensions D2—Economy and D4—Education present similar behavior in all of the clusters and range between a moderate and severe intensity.
- In the majority of the clusters, there is an absence of exclusion or a slight exclusion for the dimensions D1—Housing, D6—Isolation and D8—Social acceptance.
- The principal differences among the groups are situated in dimensions D3—Work (in which severe exclusion is predominant), D5—Health (with observed absence of exclusion or moderate exclusion, by cluster), D7—Conflict (in which there is severe exclusion or absence of exclusion, by group).
- Cluster 5 is that which shows greatest global intensity of social exclusion, followed by cluster 2 and cluster 3. The cluster that presents less intensity of exclusion is cluster 1.

**Figure 3:**
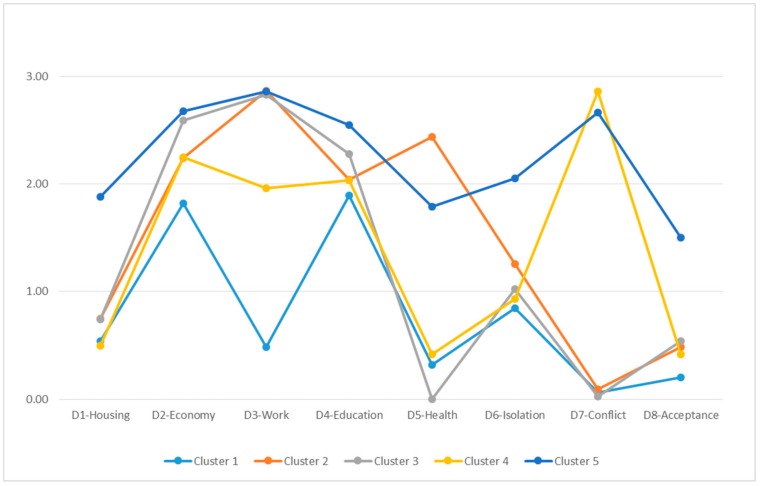
Scope and intensity of social exclusion by centers for the final clusters (five groups).

Thus, the characteristics shown in each cluster, considering the scope and intensity of exclusion in each are the following (Figure 3):

- Cluster 1. Moderate exclusion in terms of economy-education/training, with slight intensity in social relationships (isolation).
- Cluster 2. Severe exclusion in terms of work with moderate intensity in economy-education/training-health and slight exclusion in terms of housing–social relationships (isolation).
- Cluster 3: Severe exclusion in economy-work with moderate intensity in training and slight intensity in housing and in the social relationships area (isolation/social acceptance).
- Cluster 4: Severe exclusion in the area of social relationships (family and social conflict) with moderate intensity in economy-work–education/training and with slight intensity in social relationships (isolation).
- Cluster 5: Severe exclusion in economy-work-training with severe intensity or moderate in terms of housing and in the social relationships area (isolation/family or social conflict/social acceptance).

The sociodemographic characteristics of the people included in each cluster were as follows (Table 5):

## 4. Discussion

The results show that the majority of the participants are fundamentally affected by exclusion in the areas of work and employment, in the area of income level and in the area of education and training—areas that are interrelated. We observed moderate levels of exclusion in housing and in social isolation. Finally, although the dimensions with the lowest levels of exclusion are social acceptance, family and social conflict and health (over 65% of participants do not present exclusion), around a third of the users show severe or moderate exclusion.

According to the analyses carried out, the study population can be classified into five groups that are significantly different from each other and in which the scope and intensity of social exclusion varies for each dimension. With the precaution that is warranted given the exploratory character of this analysis, and without considering the process and qualitative aspects involved, the reach and scope of exclusion in the study population can be assigned for the clusters to the following trajectories established in the literature:

- Trajectory 1 “From integration to vulnerability”. This corresponds to cluster 1, characterized by moderate social exclusion in economy-education/training, with slight intensity in terms of social relationships (isolation). From the perspective of sociodemographics, the primary difference observed with respect to the rest of the clusters is that it is the group with the lowest percentage of people with Roma ethnicity (0.6%) and with the greatest percentage of people who finished their mandatory schooling (56%).
- Trajectory 2 “In permanent vulnerability”. This corresponds to cluster 4, with the following characteristics: severe exclusion in the area of social relationships (family and social conflict) with moderate intensity in terms of economy-work-education/training and with slight intensity in terms of social relationships (isolation). In comparison with the rest of the clusters, it is the second group with the lowest percentage of members of the Roma ethnicity (0.7%) and of people in an irregular legal situation or in the process of regularization (1%).
- Trajectory 3 “From integration to exclusion”. This corresponds to group 2, which is characterized by severe exclusion in terms of work with moderate intensity in economy-education/training-health and slight intensity in housing and in the social relationship area (isolation). In terms of sociodemographics, the most relevant characteristics compared to the other clusters are the following: it is the group of families with the lowest number of minors age 0 to 16 and the group that presents the greatest relative percentage of people with illness and disability (75%), with ability limitations (62%) and with the need for health care and health follow-up (81%).
- Trajectory 4 “From vulnerability to exclusion”. This corresponds to group 3, characterized by severe exclusion in economy-work with moderate intensity in education/training and slight intensity in housing and in the social relationship area (isolation/social acceptance). The primary sociodemographic difference with respect to all of the clusters is that it is the group with the greatest percentage of foreigners (50%).
- Trajectory 5 “In permanent exclusion”. This corresponds to cluster 5, characterized by severe exclusion in economy-work-education/training with severe intensity or moderate intensity in housing and in the area of social relationships (isolation, family or social conflict, social acceptance. It should be noted in terms of sociodemograhics that this group includes the greatest percentage of people with Roma ethnicity (8%), of people with an irregular legal status in the country (12%) and without completion of mandatory schooling (75%). Also, there is a relatively high percentage of people with illness and/or disability (57%) and with needs for health care and health follow-up (63%).

Finally, it should also be noted that if we consider the distribution of the population in each cluster, one of three users can be found in the trajectory that goes from vulnerability to social exclusion.

## 5. Conclusions

The results show the utility of having evidence that is empirical and rigorous regarding the characteristics of the users of social services, related to the scope and intensity of the social exclusion that affects them, and related to the typology of situations of exclusion they are subject to and their internal characteristics. In our opinion, this utility is two-fold:

- In terms of professional practice, it supports the design of intervention protocols based on evidence that can help to lay out objectives, priority action area, the typology and intensity of actions, their duration and the needed resources, etc.
- In terms of uses for organizations and policy makers, this evidence allows for the design of public policies tailored to the needs of people and the places they reside, considering the priority area of public policy, the typologies and intensity and needed resources, etc.

Certainly, in both cases, the aim is to improve professional intervention and public policies, using the evidence resulting from available data and research, in order to promote the quality of life of the users of social services. For this it is necessary that these services design and implement robust information systems regarding their users, that they systematically and periodically collect data using large samples or which is statistically significant and that can be analyzed in a rigorous way with the transfer of results to professional practice. 

In relation to the specific findings of the work, it should first be noted that the fact that one-third of the users is in the path of vulnerability to social exclusion shows the fragility of the situations of these users of social services and the need to develop differential responses from professionals and institutions to these situations.

The different trajectories identified show that social services professionals, organizations and policy makers should diversify their responses to adjust them to the characteristics of trajectories and groups, in terms of the scope and intensity of the interventions and the type of actions and resources needed. This type of information is useful for determining:

- The scope of the interventions, when orienting on the areas that are the competence of the social services in which it is necessary to act (material support, autonomy, family and social relations, etc.), and on the areas in which is necessary to connect people with other social protection systems (housing, work, education-training, health, etc.) and establish coordination systems between their professionals and organizations.
- The intensity of the necessary interventions with each group, in terms of urgency, number and periodicity of actions, considering the intensity of the exclusion according to the vital area/dimension affected.
- The type of actions required with each group: information, advice-support, educational-training, material or institutional support, advocacy, supervision-monitoring, evaluation, organizational and professional networking, internal and external coordination, etc.
- The typology of resources needed, both human and benefits, programs and services, and its priority.

Finally, although the purpose of the work was exploratory and descriptive, it is necessary to point out that the main limitation of the work is that the data, obtained from interviews with users conducted by social service workers that completed the SES-UA, need to be triangulated with other sources and types of data to make them more robust. Further work is required to deepen, qualitatively and quantitatively, in the paths of users between inclusion and exclusion to know in more detail the dynamics of the processes of exclusion and the factors underlying them.

## Figures and Tables

**Table 1:** Intensity and Scope of Social Exclusion (*n*, %).

	Level of Exclusion (Intensity)									
Dimensions (Scope)	Without Exclusion		Slight Exclusion		Moderate Exclusion		Severe Exclusion		Total	
	*n*	%	*N*	%	*n*	%	*n*	%	*n*	%
D1—Housing	569	56.4	136	13.5	201	19.9	103	10.2	1009	100.0
D2—Economy	35	3.5	155	15.4	239	23.7	580	57.5	1009	100.0
D3—Work	130	12.9	104	10.3	104	10.3	671	66.5	1009	100.0
D4—Education	40	4.0	195	19.3	329	32.6	445	44.1	1009	100.0
D5—Health	658	65.2	0	0.0	190	18.8	161	16.0	1009	100.0
D6—Isolation	327	32.4	305	30.2	250	24.8	127	12.6	1009	100.0
D7—Conflict	697	69.1	19	1.9	64	6.3	229	22.7	1009	100.0
D8—Acceptance	730	72.3	0	0.0	239	23.7	40	4.0	1009	100.0

**Table 2:** Analysis of Variance (ANOVA).

Dimensions	Cluster		Error		F	Sig.
	Quadratic Mean	df	Quadratic Mean	df		
D1—Housing	49.340	4	0.955	1004	51.675	0.000
D2—Economy	22.430	4	0.658	1004	34.101	0.000
D3—Work	202.295	4	0.391	1004	517.576	0.000
D4—Education	11.113	4	0.724	1004	15.350	0.000
D5—Health	238.165	4	0.516	1004	461.429	0.000
D6—Isolation	37.867	4	0.899	1004	42.126	0.000
D7—Conflict	378.470	4	0.132	1004	2861.990	0.000
D8—Acceptance	39.148	4	0.802	1004	48.826	0.000

**Table 3:** Number of cases in each cluster.

Cluster Number	*N*	%
1	178	17.6
2	198	19.6
3	344	34.1
4	141	14.0
5	148	14.7
Total	1009	100.0

**Table 4:** Intensity of exclusion by center in the final clusters ^1^.

Dimensions	Cluster				
	1	2	3	4	5
D1—Housing	S	S	S	∅	M
D2—Economy	M	M	SE	M	SE
D3—Work	∅	SE	SE	M	SE
D4—Education	M	M	M	M	SE
D5—Health	∅	M	∅	∅	M
D6—Isolation	S	S	S	S	M
D7—Conflict	∅	∅	∅	SE	SE
D8—Acceptance	∅	∅	S	∅	M

^1^ Note: ∅ (Without exclusion), S (Slight exclusion), M (Moderate exclusion), SE (Severe exclusion).

**Table 5:** Sociodemographic characteristics of the clusters (%, Mean and SD).

Sociodemographic Variables	Cluster 1	Cluster 2	Cluster 3	Cluster 4	Cluster 5	Total
Gender						
Woman	63.5	62.1	65.4	73.0	64.2	65.3
Man	36.5	37.9	34.6	27.0	35.8	34.7
Total	100.0	100.0	100.0	100.0	100.0	100.0
Age						
Mean (SD)	40.8 (9.4)	47.1 (11.1)	40.7 (11.0)	39.8 (10.3)	40.0 (9.0)	41.7 (10.8)
Number of members in family unit						
Mean (SD)	3.3 (1.5)	2.7 (1.4)	3.4 (1.7)	3.3 (1.5)	2.7 (1.316)	3.1 (1.6)
Number of minors age 0–16 in the family unit						
Mean (SD)	1.3 (1.2)	0.70 (1.1)	1.4 (1.2)	1.5 (1.1)	1.1 (1.4)	1.9 (1.0)
Nationality						
Spanish	63.5	73.7	49.7	65.2	62.8	61.0
Foreign	36.5	26.3	50.3	34.8	37.2	39.0
Total	100.0	100.0	100.0	100.0	100.0	100.0
Roma ethnicity						
Yes	0.6	1.5	1.8	0.7	8.1	2.3
No	99.4	98.5	98.2	99.3	91.9	97.7
Total	100.0	100.0	100.0	100.0	100.0	100.0
Legal status situation in the country						
Irregular or in the process of regularization	2.8	3.0	3.8	1.4	12.2	4.4
Regular	97.2	97.0	96.2	98.6	87.8	95.6
Total	100.0	100.0	100.0	100.0	100.0	100.0
Education level						
Has not completed mandatory schooling	43.7	57.5	59.6	59.0	75.0	58.7
Completed mandatory schooling	56.3	42.5	40.4	41.0	25.0	41.3
Total	100.0	100.0	100.0	100.0	100.0	100.0
Illness/disability						
Without illness/disability	82.1	24.7	87.1	77.1	43.2	66.2
With illness /disability	17.9	75.3	12.9	22.9	56.8	33.8
Total	100.0	100.0	100.0	100.0	100.0	100.0
Ability limitations						
Does not present	86.8	38.0	91.9	84.4	61.4	75.2
Presents (diagnosed or not)	13.2	62.0	8.1	15.6	38.6	24.8
Total	100.0	100.0	100.0	100.0	100.0	100.0
Attention and/or health follow-up						
Does not need	78.4	19.0	79.8	75.0	37.4	60.8
Needs (punctual or continuous)	21.6	81.0	20.2	25.0	62.6	39.2
Total	100.0	100.0	100.0	100.0	100.0	100.0
